# Fisetin Inhibits Periodontal Pathogen-Induced EMT in Oral Squamous Cell Carcinoma via the Wnt/β-Catenin Pathway

**DOI:** 10.3390/nu17223522

**Published:** 2025-11-11

**Authors:** Ruoyao Zhang, Hiroki Takigawa, Hugo Maruyama, Takayuki Nambu, Chiho Mashimo, Toshinori Okinaga

**Affiliations:** 1 Graduate School of Dentistry (Microbiology), Osaka Dental University, Hirakata 573-1121, Japan; lilacs416@163.com; 2 Department of Microbiology, Osaka Dental University, Hirakata 573-1121, Japan; takigawa@cc.osaka-dent.ac.jp (H.T.); nambu-t@cc.osaka-dent.ac.jp (T.N.); mashimo@cc.osaka-dent.ac.jp (C.M.)

**Keywords:** oral squamous cell carcinoma (OSCC), periodontal pathogens, epithelial–mesenchymal transition (EMT), Wnt/β-catenin

## Abstract

**Objective**: Previous reports showed that periodontopathic bacteria induce epithelial–mesenchymal transition (EMT) in oral squamous cell carcinoma (OSCC). Fisetin, a foodborne flavonoid, is reportedly associated with anticancer potential in various carcinogenic processes. This study aimed to elucidate the effects of fisetin on *Fusobacterium nucleatum*- and *Porphyromonas gingivalis*-induced EMT in OSCC cells. **Methods**: OSCC cells were co-cultured with live and heat-killed forms of *F. nucleatum* and *P. gingivalis*. The concentration of fisetin was set at 10 μM. Morphological changes in the OSCC cells were observed under a light microscope. Cell viability was measured using the Cell Counting Kit-8 assay, whereas migration was examined via wound healing. The mRNA expression of EMT-related markers was quantified using quantitative real-time polymerase chain reaction (PCR), and the expression of EMT-related markers and Wnt pathway-associated proteins was examined via Western blotting. **Results**: At a multiplicity of infection (MOI) of 300:1 for *F. nucleatum* and 100:1 for *P. gingivalis*, OSCC cell viability remained unchanged; however, wound closure rates increased significantly relative to the control. Likewise, treatment with fisetin (10 µM) did not materially alter viability; nevertheless, it attenuated promigratory effects induced by heat-killed periodontal pathogens at 3 h and 6 h. The OSCC cells exhibited EMT-like morphological changes after 6 h of co-culture with heat-killed pathogens. Consistently, reverse-transcriptase quantitative PCR and Western blot analyses showed increased expression of TWIST, ZEB1, and N-cadherin, accompanied by decreased E-cadherin expression, which was more pronounced in *F. nucleatum* than in *P. gingivalis*. However, fisetin reversed these trends. Moreover, co-culture with heat-killed pathogens markedly elevated β-catenin protein levels. In line with modulation of canonical Wnt/β-catenin signaling, fisetin and a Wnt inhibitor reduced β-catenin expression, whereas co-treatment with a Wnt agonist restored β-catenin levels in the presence of fisetin. **Conclusions**: Heat-killed *F. nucleatum* and *P. gingivalis* induced EMT in OSCC cells, with *F. nucleatum* exerting the strongest effect. Fisetin suppressed pathogen-driven EMT, at least partly via canonical Wnt/β-catenin signaling, highlighting its potential therapeutic value and warranting further investigation.

## 1. Introduction

Oral squamous cell carcinoma (OSCC) is a major head and neck malignancy characterized by high local invasiveness and frequent lymph node metastasis [1]. Diagnostic and therapeutic options have improved; nonetheless, OSCC still shows relatively poor 5-year survival, imposing a substantial burden on families and society [2,3]. A key mechanism underlying OSCC malignancy is the epithelial–mesenchymal transition (EMT), a dynamic shift in which epithelial cells forfeit polarity and cell–cell contacts while adopting mesenchymal migratory and invasive behaviors [4]. As EMT proceeds, epithelial markers such as E-cadherin decline, whereas mesenchymal markers (e.g., N-cadherin and vimentin) increase, accompanied by prometastatic transcription factor activation (e.g., *Slug*, *Snail*, *ZEB1/2*) [5]. Ultimately, this process promotes metastasis and enhances drug resistance in tumors [6,7]. Collectively, these observations show that EMT is a tractable axis for the mitigation of OSCC dissemination.

The complexity of oral microbial ecosystems provides diverse niches for bacterial colonization [8]. Among its inhabitants, periodontal pathogens, a group of microorganisms with specialized pathogenic potential [9], have been increasingly implicated in OSCC initiation and progression [10]. In particular, *Porphyromonas gingivalis* and *Fusobacterium nucleatum*, which are abundant in the subgingival plaque of patients with chronic periodontitis, destroy supporting periodontal tissues and contribute to oral carcinogenesis by inducing host immune dysregulation and promoting the malignant transformation of epithelial cells [11,12]. Building on this clinical linkage, experimental studies have indicated that these periodontal pathogens can exacerbate OSCC progression by inducing an EMT phenotype characterized by E-cadherin loss and N-cadherin upregulation [13,14]. Mechanistically, the Wnt/β-catenin pathway is a key driver of this pathogen-induced EMT process within OSCC [15]. Aberrant Wnt pathway activation by pathogens results in the transcriptional induction of EMT-promoting genes such as *ZEB1* and *Twist* [16]. Thus, pharmacologic interruption of Wnt/β-catenin signaling is a rational strategy to counteract pathogen-driven EMT and may serve as an adjunct to existing OSCC therapies.

Natural products have garnered significant attention owing to their multitarget actions and high safety profiles. Fisetin (3,3′,4′,7-tetrahydroxyflavone), a naturally occurring flavonoid that is abundant in fruits and vegetables, possesses anti-inflammatory, antioxidant, and anticancer properties [17]. Substantial preclinical evidence indicates that fisetin inhibits tumor initiation, progression, and metastasis by modulating multiple targets and pathways [18]. However, its effect on the periodontal pathogen-induced EMT in OSCC remains unclear. To further investigate the action of fisetin, co-culture models of heat-killed *F. nucleatum* and *P. gingivalis* in OSCC cells were established, and it was examined whether fisetin attenuated the pathogen-driven promigratory phenotypes and EMT. To directly test this hypothesis, we designed the following:

The effects of heat-killed *F. nucleatum* and *P. gingivalis* at multiplicities of infection (MOIs) of 100 and 300 on the migration of OSCC cells were examined. Subsequently, OSCC cell migration in the presence and absence of fisetin was quantified, the associated morphological changes were recorded, and the alterations in EMT markers were profiled. Finally, the changes in EMT markers in the presence and absence of fisetin and Wnt pathway perturbation using either an agonist or an inhibitor were assessed. Clarifying this mechanism would provide a scientific foundation for the potential application of fisetin or its analogs as adjunctive agents in OSCC therapy, particularly in patients with concomitant periodontal disease.

## 2. Materials and Methods

### 2.1. Reagents

Fisetin (purity > 96.0%, determined by HPLC) (Figure 2A) was obtained from Tokyo Chemical Industry Co., Ltd. (Tokyo, Japan). CHIR99021, a Wnt pathway agonist, and XAV939, a Wnt pathway inhibitor, were obtained from FUJIFILM Wako Pure Chemical Corporation (Osaka, Japan). Stock solutions were prepared in dimethyl sulfoxide (DMSO): fisetin (10 mM), CHIR99021 (5 mM), and XAV939 (5 mM). Aliquots were stored at −20 °C until use. Antibodies that were used included β-actin (GeneTex, Irvine, CA, USA), E-cadherin (Cell Signaling Technology, Danvers, MA, USA), ZEB1 (Proteintech, Rosemont, IL, USA), N-cadherin (#13116; Cell Signaling Technology, Danvers, MA, USA), and β-catenin (ab32572; Abcam, Cambridge, MA, USA). Horseradish peroxidase-conjugated mouse secondary antibodies against rabbit IgG were obtained from Santa Cruz Biotechnology (Dallas, TX, USA).

### 2.2. Bacterial and Cell Culture

*F. nucleatum* JCM 12990 and *P. gingivalis* ATCC 33277 were cultured on Gifu anaerobic medium (mGAM) broth agar plates (Shimadzu Diagnostics Corporation, Tokyo, Japan) and maintained anaerobically at 37 °C for 3 days. For treatment preparation, single colonies of both organisms were selected and cultured in the Gifu anaerobic medium (mGAM). The OD600 value was used as an indicator of the desired bacterial concentration (CFU/mL). Heat-killed bacteria were prepared by incubating bacterial suspensions for 15 min at 100 °C. The human OSCC cell line squamous cell carcinoma (SAS) (Japanese Collection of Research Bioresources Cell Bank, Osaka, Japan), originally established from a poorly differentiated tongue squamous cell carcinoma, was used in this study. Culture conditions for SAS cells were RPMI-1640 medium (Nacalai Tesque, Kyoto, Japan) with 10% fetal bovine serum (FBS, Gibco, Thermo Fisher Scientific, Tokyo, Japan) and 1% penicillin–streptomycin. Cells were maintained at 37 °C under 5% CO_2_ and subcultured at 90% confluence every alternate day.

### 2.3. Cell Viability Assay

SAS cells (1 × 10^4^ cells/well) were seeded into 96-well plates and allowed to adhere overnight in RPMI1640 medium containing only 10% FBS to allow cell attachment. On the day after seeding, cells were exposed to fisetin (5–20 μM), Wnt pathway inhibitor XAV939 (5 μM), or Wnt pathway agonist CHIR99021 (5 μM). DMSO (0.1%) served as the control for vehicle treatment. For bacterial stimulation, cells were treated with two oral pathogens, *F. nucleatum* and *P. gingivalis*, in either live or heat-inactivated form, at infection ratios of 100:1 and 300:1. Cell viability was evaluated using Cell Counting Kit-8 (CCK-8; Dojindo Laboratories, Kumamoto, Japan) following the manufacturer’s protocol (https://www.dojindo.com/manual/CK04/, accessed on 6 November 2025). After a 24 h culture period, 10 μL CCK-8 reagent was dispensed into each well, and incubation was continued for 2 h. Optical density was measured at 450 nm using a SpectraMax M5 multimode microplate reader (Molecular Devices, San Jose, CA, USA).

### 2.4. Wound-Healing Assay

Approximately 8 × 10^5^ cells were seeded in 6-well plates. When the monolayer reached 90% confluence, a linear scratch was generated with a sterile 1000 μL pipette tip [19]. After washing twice with phosphate-buffered saline (PBS), cells were treated with *F. nucleatum*, heat-killed *F. nucleatum*, and heat-killed *P. gingivalis* at an MOI of 100:1 and 300:1; the cells were also treated with fisetin at 10 μM and maintained in the RPMI-1640 medium. Cell migration was evaluated by monitoring the wounded area at the indicated time points [20]. An OLYMPUS CKX53 Cell Culture Microscope (Olympus Co., Tokyo, Japan) was used to acquire images. The wound closure percentage was quantified using ImageJ software (version 1.53t, National Institutes of Health, Bethesda, MD, USA) using the formula: wound closure (%) = [1 − (gap area/average initial gap area) × 100%].

### 2.5. Reverse Transcription Quantitative Real-Time Polymerase Chain Reaction

Total cellular RNA was extracted using the RNeasy Mini Kit (QIAGEN, Hilden, Germany) following the manufacturer’s protocol (https://www.qiagen.com/us/resources/resourcedetail?id=f646813a-efbb-4672-9ae3-e665b3045b2b&lang=en, accessed on 6 November 2025). The purity and concentration of RNA were quantified with a NanoDrop ND-1000 spectrophotometer (Thermo Fisher Scientific, Waltham, MA, USA). Reverse transcription was performed using the ReverTra Ace qPCR RT Master Mix (TOYOBO, Osaka, Japan) to obtain complementary DNA (cDNA). Reverse transcription quantitative real-time polymerase chain reaction (RT-qPCR) was subsequently performed on a StepOnePlus Real-Time PCR System (Applied Biosystems, Foster City, CA, USA) using THUNDERBIRD Next SYBR qPCR Mix (TOYOBO). The relative expression levels of target genes were calculated using the 2^−ΔΔCt^ method [21], normalized to β-actin, and presented as fold changes relative to the control. The primer sequences used in this study are listed in Table 1.

### 2.6. Western Blotting

SAS cells were rinsed with PBS to eliminate unattached and nonviable cells. The remaining adherent cells were collected and lysed using radioimmunoprecipitation assay buffer (Nacalai Tesque, Kyoto, Japan). Following the manufacturer’s protocol (https://www.takarabio.com/documents/User%20Manual/T9300A/T9300A_UM.pdf, accessed on 6 November 2025), a Bicinchoninic Acid Protein Assay Kit (Takara Bio, Kyoto, Japan) was used to quantify total protein. Equivalent quantities of protein samples were subsequently combined with loading buffer (FUJIFILM Wako Pure Chemical Co., Tokyo, Japan), separated with sodium dodecyl sulfate–polyacrylamide gel electrophoresis (7.5% gel), and electroblotted onto polyvinylidene difluoride membranes (0.22 μm). At room temperature, Blocking One solution (Nacalai Tesque) was used to block the membranes for 1 h, which were subsequently incubated for 16 h at 4 °C with the appropriate primary antibodies. After three washes, the membranes were incubated with the appropriate secondary antibodies for 1 h. Enhanced chemiluminescence Select Western Blotting Detection Reagent (Cytiva, Logan, UT, USA) was used to detect protein signals, and chemiluminescent images were captured using the ChemiDoc imaging platform (Bio-Rad, Hercules, CA, USA). β-actin was used as the endogenous reference to normalize protein loading [22].

### 2.7. Statistical Analysis

Statistical analyses were performed using SPSS software (version 22.0). Graphs were plotted using GraphPad Prism (version 9.4.1). Each experiment was independently repeated at least three times, and results are expressed as the mean ± standard deviation (SD). Differences among groups were analyzed using one-way analysis of variance, with statistical significance set at *p* ≤ 0.05.

## 3. Results

### 3.1. Heat-Killed F. nucleatum and P. gingivalis Promote OSCC Cell Migration

**Figure 1:**
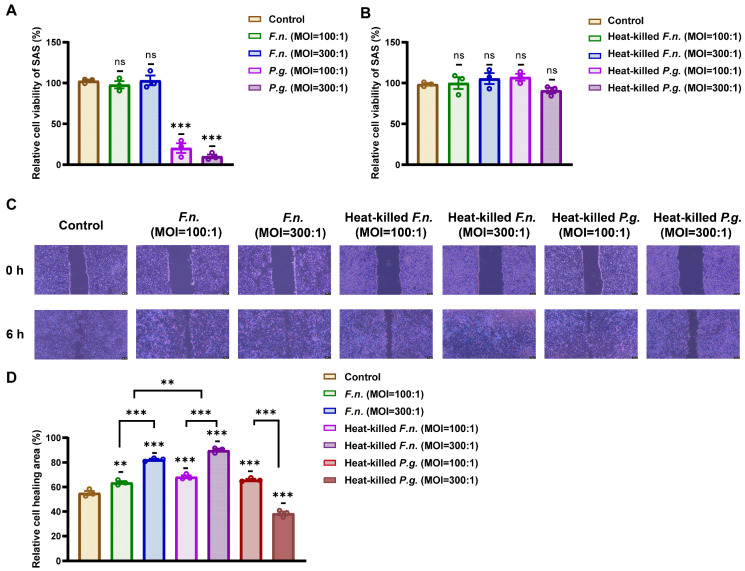
Heat-killed *F. nucleatum* and *P. gingivalis* increase oral squamous cell carcinoma cell migration under conditions that maintain viability. (**A**,**B**) Cell viability of squamous cell carcinoma cells after co-culture with live or heat-killed *F. nucleatum* or *P. gingivalis*, assessed using the Cell Counting Kit-8 assay. (**C**) Shown are representative wound-healing images captured post-scratch. Scale bar: 200 μm. (**D**) Quantification of wound closure percentage shows enhanced migration with heat-killed exposure of *F. nucleatum*, producing a stronger effect than *P. gingivalis*. Data are shown as mean ± SD (n = 3). ** *p* < 0.01, and *** *p* < 0.001, compared with that of the control or between indicated groups.

First, to determine appropriate conditions for bacterial stimulation, OSCC cell viability was evaluated after co-culture with live or heat-killed *F. nucleatum* and *P. gingivalis* at MOIs of 100:1 and 300:1. Figure 1A,B show that treatment with live *P. gingivalis* significantly reduced cell viability. Conversely, heat-killed *F. nucleatum* and *P. gingivalis* and live *F. nucleatum* did not exhibit notable cytotoxicity compared with that of the control. Owing to the significant cytotoxicity of live *P. gingivalis*, this condition was excluded from subsequent experiments.

Next, a wound-healing assay was performed to evaluate cell migration following exposure to heat-killed bacteria. OSCC cells were treated with *F. nucleatum* and heat-killed *F. nucleatum* and *P. gingivalis* for 6 h, and the effects of these treatments on their cell migration were investigated using a scratch assay. Figure 1C,D show that heat-killed *F. nucleatum* at MOIs of 300:1 and 100:1, respectively, and heat-killed *P. gingivalis* at an MOI of 100:1 significantly increased wound closure relative to the control. These conditions were selected for further analysis. Among the tested MOIs, heat-killed *P. gingivalis* at a ratio of 100:1 exhibited the most consistent enhancement of cell migration without compromising cell viability. Higher MOIs (e.g., 200:1 or 300:1) were initially tested; nevertheless, they did not result in a proportional increase in migratory behavior and occasionally showed slight cytotoxic effects. Therefore, an MOI of 100:1 (heat-killed *P. gingivalis*) was selected as the optimal non-cytotoxic condition for the subsequent assays.

### 3.2. Fisetin Inhibits OSCC Cell Migration Induced by Heat-Killed Periodontal Pathogens

**Figure 2:**
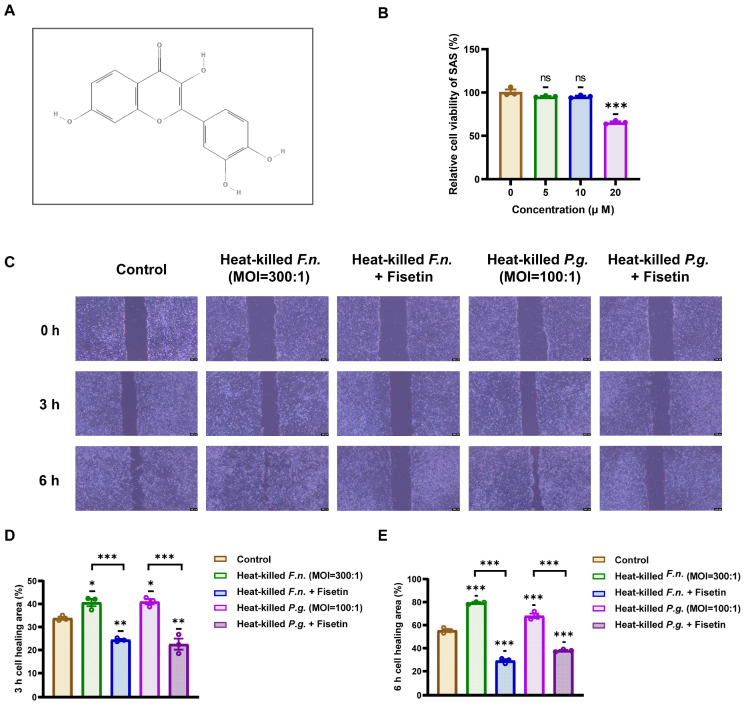
Fisetin reduces periodontal pathogen-induced migration of oral squamous cell carcinoma cells without compromising viability. (**A**) Chemical structure of fisetin. (**B**) The viability of squamous cell carcinoma cells treated with fisetin (5, 10, or 20 μM) was determined using a CCK-8 assay. (**C**) Representative wound-healing images for cells exposed to heat-killed *F. nucleatum* or *P. gingivalis* with or without fisetin (10 μM). Scale bar: 200 μm. (**D**,**E**) Quantification of wound closure percentage at 3 h and 6 h demonstrates that fisetin attenuates pathogen-induced migration. Data are shown as mean ± SD (n = 3). * *p* < 0.05, ** *p* < 0.01, and *** *p* < 0.001, compared with that of the control or between indicated groups.

The chemical structure of fisetin (3,3′,4′,7-tetrahydroxyflavone) is shown in Figure 2A. Before evaluating its functional effects, fisetin was assessed for cytotoxicity in OSCC cells. As shown in Figure 2B, treatment with 10 μM fisetin did not significantly affect the viability of OSCC cells compared with that of the untreated control, as determined via the CCK-8 assay. This observation showed that the selected concentration was non-toxic and suitable for further functional analysis.

Next, the effect of fisetin on cell migration induced by heat-killed *F. nucleatum* and *P. gingivalis* was assessed using a wound-healing assay. Co-cultures of OSCC cells with either pathogen were maintained for 3 and 6 h in the presence or absence of fisetin. Figure 2C,D show that treatment with either heat-killed *F. nucleatum* or *P. gingivalis* alone significantly enhanced the wound closure rate relative to that of the control, consistent with their promigratory effects. Notably, co-treatment with fisetin markedly attenuated this migration-promoting effect, as evidenced by a significantly reduced wound closure percentage at both time points. These findings indicated that fisetin effectively suppressed the migration-promoting effect of periodontal pathogens in OSCC cells, independent of cytotoxicity.

### 3.3. Heat-Killed Forms of F. nucleatum and P. gingivalis Can Induce the EMT Process in OSCC Cells, but Fisetin Inhibits This Effect

**Figure 3:**
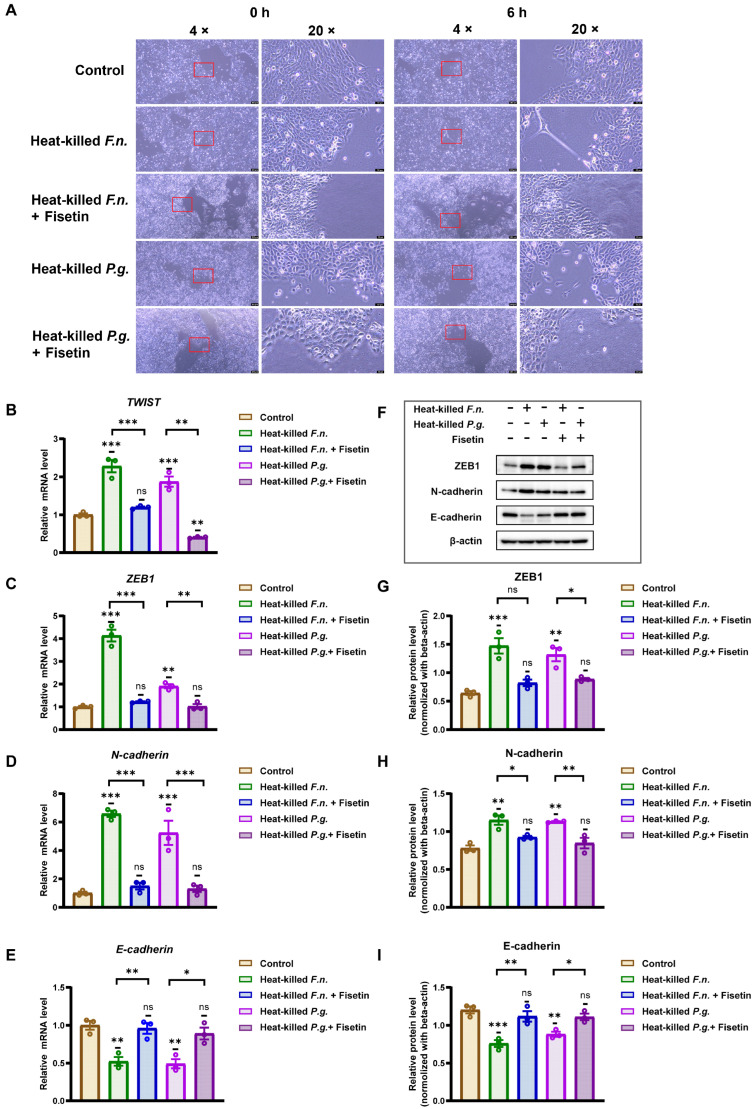
Fisetin attenuates epithelial–mesenchymal transition (EMT) induced by heat-killed periodontal pathogens in oral squamous cell carcinoma (OSCC) cells. (**A**) Morphological changes in OSCC cells observed using light microscopy, co-cultured with heat-killed *F. nucleatum* or *P. gingivalis*. Scale bar: 200 μm (left) and 50 μm (right). (**B**–**E**) Pathogen exposure decreased E-cadherin expression and increased that of TWIST, ZEB1, and N-cadherin; fisetin (10 μM) blunted these changes relative to the corresponding pathogen-alone group. (**F**) EMT-related protein expression was evaluated using Western blotting. (**G**–**I**) Quantification of protein expression levels showing restoration of E-cadherin expression and reduction in that of ZEB1 and N-cadherin in fisetin co-treatment versus pathogen-alone conditions. Data are shown as mean ± SD (n = 3). * *p* < 0.05, ** *p* < 0.01, and *** *p* < 0.001, compared with that of the control or between indicated groups.

To further examine the effects of fisetin and heat-killed *F. nucleatum* and *P. gingivalis* on OSCC cells, the cells were co-cultured with either pathogen for 6 h with or without fisetin, and morphological changes were observed. As shown in Figure 3A, the control OSCC cells formed adherent colonies with typical epithelioid and polygonal morphologies. In contrast, cells treated with heat-killed *F. nucleatum* or *P. gingivalis* displayed elongated shapes and slender protrusions. However, when co-treated with fisetin, the cells largely retained an epithelial-like morphology.

These morphological changes, together with the enhanced migratory behavior, suggest the potential involvement of EMT. To confirm this observation, RT-qPCR was used to assess the mRNA levels of EMT-associated genes. As shown in Figure 3B–E, RT-PCR analysis revealed that treatment with heat-killed *F. nucleatum* or *P. gingivalis* upregulated the expression of transcription factors (*TWIST*, *ZEB1*) and *N-cadherin*, whereas it downregulated *E-cadherin* expression. Notably, heat-killed *F. nucleatum* induced stronger EMT-associated transcriptional changes than heat-killed *P. gingivalis*. Co-treatment with fisetin inhibited these changes: compared with that of the heat-killed bacteria-only condition, expression levels of *TWIST*, *ZEB1*, and *N-cadherin* decreased, whereas *E-cadherin* expression was elevated under co-treatment conditions with fisetin.

Western blotting further supported these findings (Figure 3F–I). Both heat-killed *F. nucleatum* and *P. gingivalis* increased the protein expression of ZEB1 and N-cadherin while reducing E-cadherin expression relative to that of the control. Consistent with the RT-PCR results, heat-killed *F. nucleatum* exerted a more pronounced effect than *P. gingivalis* did. Importantly, fisetin reduced ZEB1 and N-cadherin protein levels, whereas it restored E-cadherin expression. These results confirm that fisetin suppresses the EMT induced by heat-killed *F. nucleatum* and *P. gingivalis* in OSCC cells.

### 3.4. Fisetin Inhibits Periodontal Pathogen-Induced EMT Processes in OSCC via the Wnt/β-Catenin Pathway

**Figure 4:**
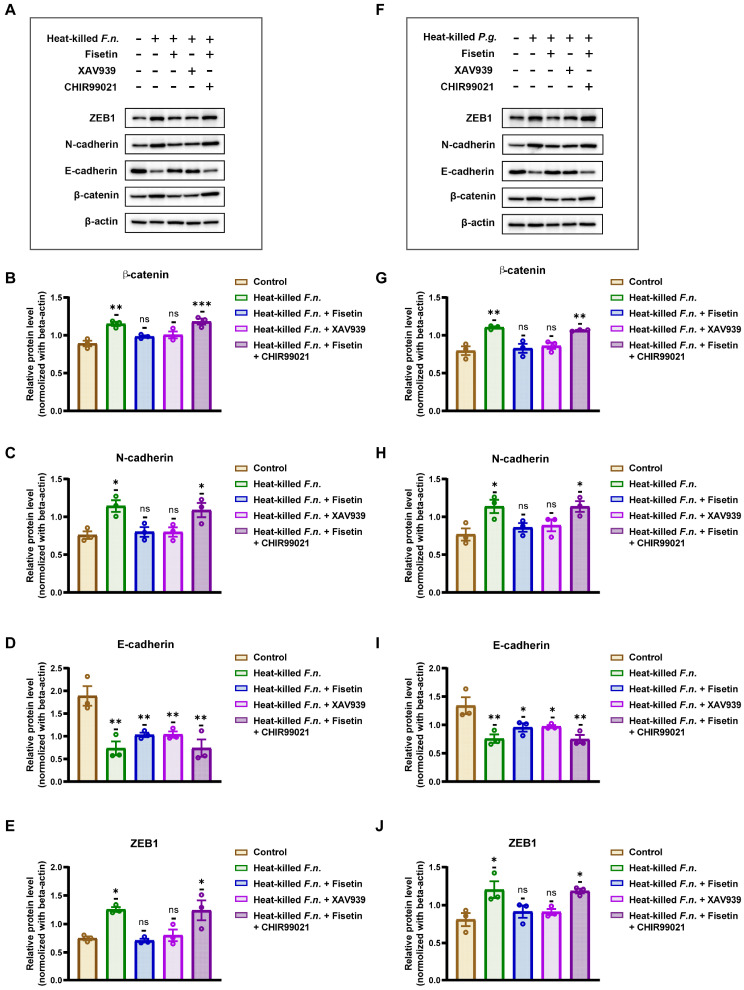
Fisetin counteracts heat-killed periodontal pathogen-induced epithelial–mesenchymal transition (EMT), consistent with modulation of canonical Wnt/β-catenin signaling. (**A**,**F**) Western blot analysis of EMT-related proteins expression in OSCC cells co-cultured with heat-killed *F. nucleatum or P. gingivalis*, with or without fisetin (10 μM), Wnt inhibitor XAV939, or Wnt agonist CHIR99021. (**B**–**E**,**G**–**J**) Western blotting analysis and quantification results of EMT-associated proteins. Key result: heat-killed bacteria decrease the expression of E-cadherin and increase that of ZEB1, N-cadherin, and β-catenin; fisetin and XAV939 attenuate these changes, whereas CHIR99021 restores them in the presence of fisetin, supporting Wnt/β-catenin involvement. Data are shown as mean ± SD (n = 3). * *p* < 0.05, ** *p* < 0.01, and *** *p* < 0.001, compared with that of the control group.

Accumulating evidence suggests that the Wnt pathway is pivotal for the induction of EMT. To clarify the mechanisms by which this pathway regulates the EMT, specific Wnt agonists and inhibitors were used, and the expression of EMT-associated proteins was analyzed. As shown in Figure 4A,B,F,G, co-culture of OSCC cells with heat-killed *F. nucleatum* or *P. gingivalis* markedly increased β-catenin protein levels, a key downstream molecule of the Wnt pathway, indicating activation of the canonical Wnt/β-catenin pathway. Conversely, co-treatment with fisetin abolished this effect, and β-catenin expression remained comparable to that of the control. Similar results were obtained using XAV939, a Wnt pathway inhibitor. Notably, when fisetin was combined with CHIR99021, a Wnt agonist, β-catenin expression was restored, confirming reactivation of the Wnt pathway.

Figure 4C–E,G–J show that the EMT process in OSCC was positively correlated with Wnt/β-catenin pathway activity. Exposure to heat-killed *F. nucleatum* and *P. gingivalis* or co-treatment with fisetin and a Wnt agonist resulted in pathway activation, accompanied by increased expression of ZEB1 and N-cadherin and decreased E-cadherin expression. Conversely, co-treatment with fisetin or a Wnt inhibitor suppressed the Wnt pathway and blocked EMT progression. Under these conditions, ZEB1 and N-cadherin expression with fisetin co-treatment did not significantly differ from that of the control, whereas E-cadherin expression, although lower than that in the control group, was significantly higher than that in the two pathogen- or agonist-treated groups. These findings indicate that the Wnt/β-catenin pathway is a key regulator of EMT in OSCC cells induced by heat-killed *F. nucleatum* and *P. gingivalis*. Fisetin effectively inhibited the activation of this pathway, thereby suppressing pathogen-induced EMT and limiting OSCC cell migration and invasion.

## 4. Discussion

The present study shows that heat-killed *F. nucleatum* and *P. gingivalis* can induce EMT in OSCC cells. Importantly, the natural flavonoid fisetin suppressed pathogen-driven EMT, at least partly via canonical Wnt/β-catenin signaling.

Substantial evidence from recent studies has shown a strong association between periodontal pathogens, notably *F. nucleatum and P. gingivalis*, and OSCC onset and progression [10,12,23]. Even after heat inactivation, these bacteria promote OSCC cell migration and trigger the EMT process [14,19,24]. Heat-killed *F. nucleatum* and *P. gingivalis* upregulate pro-inflammatory mediators (TGF-β1, EGF, and TNF-α) and activate the transcription factors Snail and TWIST, thereby repressing E-cadherin expression [25]. TWIST further enhances β-catenin-dependent transcription [26], collectively reinforcing EMT programs in OSCC cells. While the data show that heat-killed periodontal pathogens can induce EMT, the precise mechanisms involved have not been fully elucidated. The results indicate that heat-killed *F. nucleatum* and *P. gingivalis* induce EMT in OSCC cells, which is characterized by reduced E-cadherin and increased N-cadherin expressions, respectively, and upregulation of the transcription factor ZEB1.

Notably, these results showed that heat-killed *F. nucleatum* exhibited a stronger pro-EMT effect than heat-killed *P. gingivalis*, likely because of the unique adhesion molecular structure and the multipathway activation capacity of *F. nucleatum*. It uses its virulence factor FadA adhesin to bind host E-cadherin, directly disrupting intercellular junctions and activating the Wnt/β-catenin pathway, consequently inducing EMT-related gene expression [27]. Notably, FadA is a heat-resistant factor, and its structural stability allows it to remain functional even after heat inactivation [28]. In contrast, the principal virulence determinants of *P. gingivalis* are gingipains (e.g., Rgp and Kgp), which depend on proteolytic activity to degrade host intercellular proteins (e.g., E-cadherin) [29]. Heat inactivation abolishes this enzymatic activity, thereby reducing the capacity to promote EMT. In addition, the lipopolysaccharide (LPS) of *F. nucleatum* primarily activates the JNK and NF-κB pathways via toll-like receptor 4 (TLR4), prompting the secretion of cytokines such as interleukin (IL)-6, IL-8, and TGF-β [30,31]. These inflammatory and growth factors further promote EMT [32,33,34]. Heat inactivation did not destroy the LPS of *F. nucleatum*, allowing it to continuously exert its stimulatory effects. In contrast, the LPS of *P. gingivalis* contains multiple lipid A structures and preferentially activates epithelial cells via the TLR2 pathway, eliciting a comparatively weaker inflammatory response [35,36]. Furthermore, the immunostimulatory activity of heat-killed *P. gingivalis* may be reduced, diminishing its capacity to induce EMT [37,38]. Collectively, these mechanisms explain why heat-killed *F. nucleatum* retains a stronger ability to promote EMT than that of heat-killed *P. gingivalis*.

Fisetin, enriched in strawberries, apples, and onions [39], has shown antitumor effects in lung, breast, colorectal, and other cancers by inhibiting proliferation, promoting apoptosis, and suppressing migration and invasion [40,41,42]. The antitumor effects of fisetin are mediated through the regulation of several pathways, including the PI3K/Akt/mTOR, MAPK/ERK, and Wnt/β-catenin pathways [18]. Fisetin has also been shown to inhibit EMT by upregulating the epithelial marker E-cadherin and downregulating mesenchymal markers, including vimentin and N-cadherin, in various tumor cells [40,43,44]. Evidence for its effects on the Wnt/β-catenin pathway remains relatively limited; however, melanoma cell studies showed that fisetin treatment reduces GSK3β phosphorylation, decreases β-catenin stability, and thereby suppresses the EMT phenotype [45]. Similarly, in pancreatic cancer models, fisetin downregulates the expression of β-catenin, inhibiting tumor growth [46]. Consistent with these findings, the present study shows that fisetin inhibits the Wnt/β-catenin pathway, thus obstructing the EMT process induced by heat-killed *F. nucleatum* and *P. gingivalis* in OSCC cells. Collectively, these findings indicate that fisetin, as a multitarget natural small molecule, has significant potential for inhibiting tumor metastasis and reversing EMT by downregulating the Wnt pathway and EMT effector molecules. To our knowledge, this is the first study showing that fisetin inhibits periodontal pathogen-induced EMT in OSCC cells via the Wnt/β-catenin pathway.

Periodontal pathogen stimulation in this OSCC cell model significantly elevated β-catenin expression after exposure to *P. gingivalis* and *F. nucleatum*. Concurrently, the cells exhibited characteristic molecular alterations associated with EMT, including the downregulation of E-cadherin expression and significant upregulation of the expressions of N-cadherin and ZEB1. A transition in cell morphology was observed with OSCC cells, adopting mesenchymal-like traits from an epithelial-like phenotype. Co-treatment with fisetin decreased β-catenin levels and inhibited EMT, an effect reversed by a Wnt agonist. These findings indicate that *P. gingivalis* and *F. nucleatum* induce EMT primarily through the Wnt/β-catenin pathway and that fisetin inhibits this pathogen-induced EMT by antagonizing Wnt signaling. In summary, fisetin restores the epithelial phenotype of cells by correcting pathogen-induced dysregulation of the Wnt/β-catenin pathway. This study provides mechanistic evidence that the Wnt/β-catenin pathway is central to periodontal pathogen-associated EMT in OSCC and establishes fisetin as an effective inhibitor of this process. This finding indicates that targeting this pathway may represent a promising strategy to suppress microbially driven tumor progression [45,46,47]. Based on these findings, we propose the following molecular mechanisms.

Infection-driven EMT: *P. gingivalis* and *F. nucleatum* activate the Wnt/β-catenin pathway through adhesion or the use of virulence factors, driving EMT and enhancing OSCC cell migration and invasion. Targeted Intervention: Fisetin disrupts β-catenin signaling and partially restores epithelial characteristics, thereby blocking EMT progression. Despite its potential, the clinical application of fisetin remains challenging. The natural flavonoid fisetin is considered safe; however, its poor solubility and low oral bioavailability make it difficult to achieve plasma concentrations comparable to effective in vitro doses using conventional administration. Novel formulations such as nanocapsules or liposomes have been explored in current research to enhance the absorption rate and antitumor efficacy of fisetin [48,49]. Moreover, only changes in core protein levels within the Wnt/β-catenin pathway were evaluated in this study. Future studies should incorporate functional Wnt readouts, including β-catenin nuclear translocation assays, T-cell factor/lymphoid enhancer-binding factor (TCF/LEF) reporter assays, and target gene expression. Given the intricate crosstalk between periodontal pathogens and the host immune microenvironment, further studies are required to examine the anti-inflammatory and immunomodulatory effects of fisetin. In addition, possible synergistic effects with antibiotics, chemotherapeutic agents, or targeted therapies, as well as their impact on the oral microbiota, require further investigation. Collectively, these findings suggest that fisetin is a promising adjuvant for oral cancer complicated by periodontal infections.

## 5. Conclusions

Heat-killed *F. nucleatum* and *P. gingivalis* promote migration and induce EMT in OSCC cells, with *F. nucleatum* consistently exerting a stronger effect than that of *P. gingivalis*. EMT markers shifted accordingly: E-cadherin expression decreased, whereas the expression levels of N-cadherin and ZEB1 increased at the mRNA and protein levels. These phenotypes were accompanied by elevated β-catenin expression, consistent with the activation of canonical Wnt/β-catenin signaling. Fisetin significantly curtailed pathogen-induced migration and partially reversed EMT marker changes (increased E-cadherin expression and decreased the expression of N-cadherin and ZEB1), together with a reduction in β-catenin expression. Overall, the data indicate that *F. nucleatum* is a stronger EMT driver than *P. gingivalis* in OSCC and that fisetin inhibits these effects at least partly by modulating canonical Wnt/β-catenin signaling. Clinically, these findings suggest that fisetin is a plausible adjunct for suppressing microbe-driven prometastatic programs in OSCC. Future studies should integrate functional Wnt readouts (β-catenin nuclear translocation, TCF/LEF reporter assays, and target gene expression) and optimize dosing and delivery (e.g., nanoformulations or oral/topical approaches tailored to the periodontal microenvironment) to facilitate translation.

## Figures and Tables

**Table 1:** Primer used for RT-qPCR.

Gene Symbol	Forward 5′–3′	Reverse 5′–3′
*β-actin*	CATGTACGTTGCTATCCAGGC	CTCCTTAATGTCACGCACGAT
*E-cadherin*	CGAGAGCTACACGTTCACGG	GGGTGTCGAGGGAAAAATAGG
*N-cadherin*	TCAGGCGTCTGTAGAGGCTT	ATGCACATCCTTCGATAAGACTG
*ZEB1*	CACATGCGATTACATTCTGGAG	CGTGCTCATTCGAGAGGATT
*TWIST*	TGCGGAAGATCATCCCCACG	GCTGCAGCTTGCCATCTTGGA

## Data Availability

The original contributions presented in the study are included in the article, and further inquiries can be directed to the corresponding authors.
